# Proof of Concept in Assignment of Within-Subject Variability During Virtual Bioequivalence Studies: Propagation of Intra-Subject Variation in Gastrointestinal Physiology Using Physiologically Based Pharmacokinetic Modeling

**DOI:** 10.1208/s12248-021-00672-z

**Published:** 2022-01-05

**Authors:** Margareta Bego, Nikunjkumar Patel, Rodrigo Cristofoletti, Amin Rostami-Hodjegan

**Affiliations:** 1 Agency for Medicinal Products and Medical Devices (HALMED), Zagreb, Croatia; 2 Centre for Applied Pharmacokinetic Research (CAPKR), School of Health Sciences, University of Manchester, Stopford Building, Oxford Road, Manchester, M13 9PL UK; 3 Certara UK Limited, Simcyp Division, 1 Concourse Way, Sheffield, S1 2BJ UK; 4 Center for Pharmacometrics and Systems Pharmacology, Department of Pharmaceutics, College of Pharmacy, University of Florida, Orlando, Florida USA

**Keywords:** within-subject variability, intra-subject variation, virtual bioequivalence, physiology-based pharmacokinetics

## Abstract

While the concept of ‘Virtual Bioequivalence’ (VBE) using a combination of modelling, *in vitro* tests and integration of pre-existing data on systems and drugs is growing from its infancy, building confidence on VBE outcomes requires demonstration of its ability not only in predicting formulation-dependent systemic exposure but also the expected degree of population variability. The concept of variation influencing the outcome of BE, despite being hidden with the cross-over nature of common BE studies, becomes evident when dealing with the acceptance criteria that consider the 90% confidence interval (CI) around the relative bioavailability. Hence, clinical studies comparing a reference product against itself may fail due to within-subject variations associated with the two occasions that the individual receives the same formulation. In this proof-of-concept study, we offer strategies to capture the most realistic predictions of CI around the pharmacokinetic parameters by propagating physiological variations through physiologically based pharmacokinetic modelling. The exercise indicates feasibility of the approach based on comparisons made between the simulated and observed WSV of pharmacokinetic parameters tested for a clinical bioequivalence case study. However, it also indicates that capturing WSV of a large array of physiological parameters using backward translation modelling from repeated BE studies of reference products would require a diverse set of drugs and formulations. The current case study of delayed-release formulation of posaconazole was able to declare certain combinations of WSV of physiological parameters as ‘not plausible’. The eliminated sets of WSV values would be applicable to PBPK models of other drugs and formulations.

**Supplementary Information:**

The online version contains supplementary material available at 10.1208/s12248-021-00672-z.

**Figure:** Graphical Abstract

## INTRODUCTION

In the last two decades, the growth in applications of physiologically based pharmacokinetics (PBPK) has been over 10-fold greater than the general subject matter of pharmacokinetics itself (1). One of the emerging applications of the PBPK models is the conduct of virtual bioequivalence (VBE) studies (2). This is associated with the advancements in mechanistic representation of oral drug absorption that enables assessment of relative bioavailability between two formulations in the same group of subjects (3, 4) or same formulation under different conditions (5, 6) or in different populations (7). Using PBPK modelling for biopharmaceutics analysis has transitioned from ‘academic nicety to regulatory necessity’ over the last decade (8). However, the VBE concept is still in its infancy (3, 7, 9–11) and successful regulatory applications of VBE trials are still sparse in public literature (12, 13).

Clinical bioequivalence (BE) studies are designed to demonstrate similarity in the systemic exposure (*C*_max_, *t*_max_ and *AUC*) of two products containing the same active substance. Although parallel design studies might be used for the purpose of establishing BE under certain circumstances, in particular with corrections for the elimination differences in the two parallel groups (14, 15), BE studies are most often carried out using a cross-over design where each subject is administered both reference and test drug. This reduces the variability coming from different sources other than formulation-related, especially under the tightly standardized protocols which reduce the sample size (and the cost) for BE studies.

**Figure 1:**
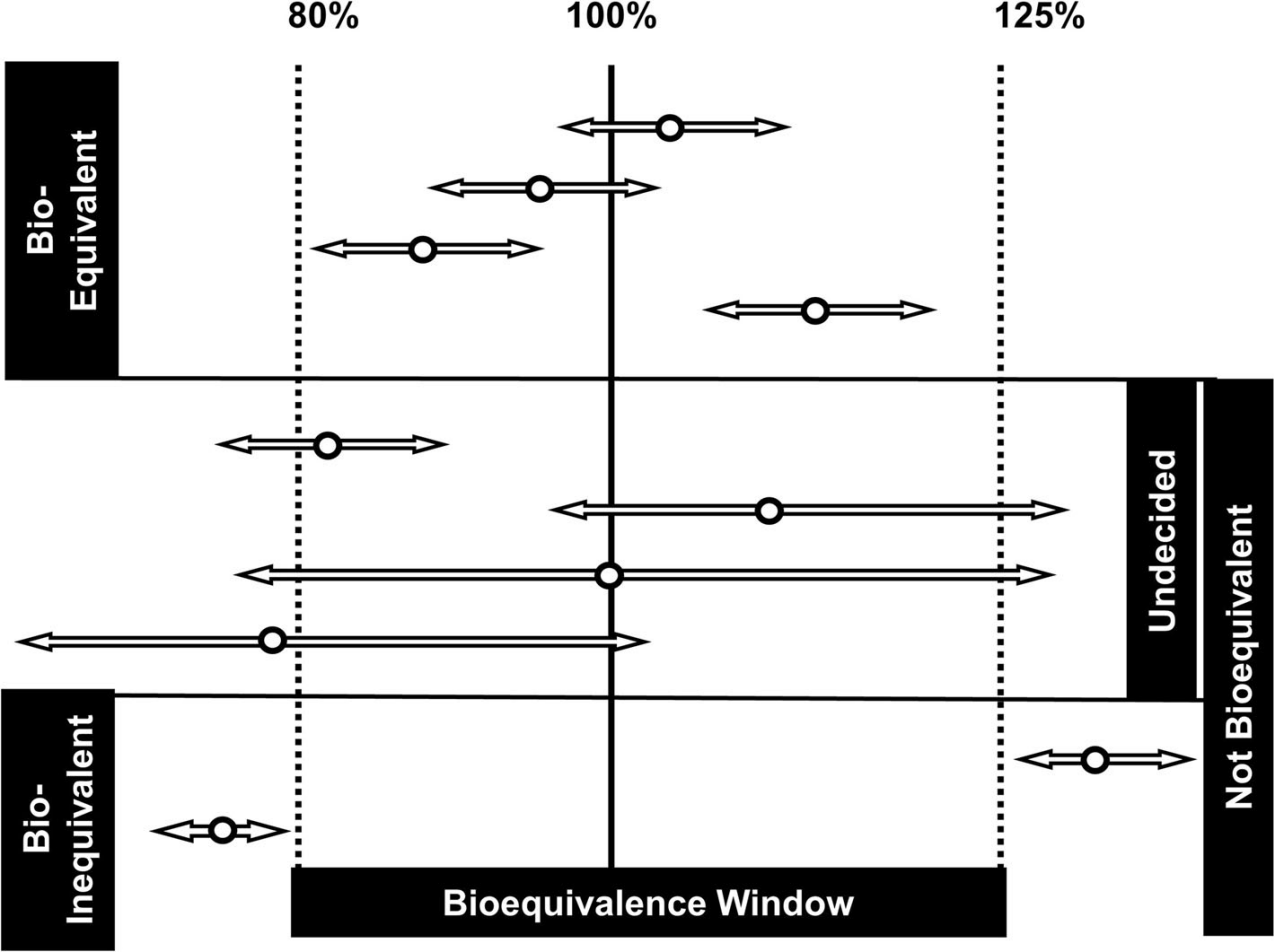
Graphical representation of current BE window and the variety of outcomes that are possible following the assessment of 90% CI around the relative bioavailability for a given marker of BE

Even though between-subject variability (BSV) can be avoided by cross-over study design, due to within-subject variability (WSV), calculated 90% confidence intervals (CI) around the average relative bioavailability may not allow the conclusion of BE to be reached when they fall outside the accepted window (see Figure 1). The amplitude of CIs in BE studies depends not only on the number of enrolled subjects, but also on the WSV in the rate and extent of bioavailability that is determined by the drug as well as formulation attributes and their interplay with the WSV in physiology, particularly those of GI tract.

A category of drugs is designated ‘highly variable’ since the measures of systemic exposures (*C*_max_and/or*AUC*) under the repeat administration to the same individual in a different period produced a coefficient of variation (CV) >30% (16, 17). A retrospective analysis of generic drug applications at the US FDA has shown that major sources of the observed intra-subject variability (also known as within-subject variability [WSV] or as inter-occasion variability [IOV], even the latter may not be considered fully exchangeable) were drug properties related to absorption. These included low aqueous solubility, low oral bioavailability, high acid lability, high lipophilicity and the extensive gut or first-pass metabolism (18).

The confidence in the VBE outcomes and hence general acceptance will greatly increase if such studies can capture the observed WSV mechanistically. In other words, VBE should minimize false positive or false negative results to allow more confidence in utilizing such advanced modelling techniques in drug development and regulatory decision-making. False positive results may arise from not considering WSV, which underestimates the amplitude of estimated CI around the geometric mean ratios of PK metrics. In turn, false negative results may occur in response to propagating inflated WSV coefficients through simulations.

In a simplest form, WSV can be included in VBE trials empirically based on the *post hoc* assignment of clinically obtained WSV coefficients from previous BE studies or prior knowledge to the simulated mean BE metrics (*C*_max_ and *AUC*) (9). Despite its simplicity, this approach generally assumes similar WSV for test and reference formulations and lacks utilization of full potential of mechanistic PBPK models in VBE assessments. Moreover, the strategy requires existence of previous replicate design clinical studies (whether full or partial) to estimate WSV of PK measures for the products. Replicate designs are not the most common BE studies. Alternatively, a mechanistic propagation of WSV in the system parameters through simulations of each product emerges as a promising approach (9). However, such strategy depends on the knowledge of mechanistic understanding of absorption as well as information on WSV of the attributes of GI tract that can impact the PK of the drug and formulation(s) of interest.

While the WSV of some GI tract parameters have been unveiled by specialized studies measuring such parameters in more than one occasion in healthy volunteers (19–21), the WSV for most of the GI parameters and their co-variations are not known (22). Some investigators have used the conservative approach by assuming the WSV in these parameters to be similar to the respective BSV which can likely be the worst-case scenario in terms of impact of WSV on VBE outcome (3). Such strategy can likely increase the risk of false negative results and thus, the sponsor risk. On the other hand, such a conservative WSV worst-case scenario approach could be useful from the risk assessment perspective by the regulatory agencies. Understanding and simulating realistic WSV is very important for future implementation of VBE approach as the conservative approach above is highly prone to false negative results limiting its utility for highly variable drugs and/or drug products.

Several reports have highlighted the need for better handling and estimation of WSV (4, 12, 22–24). However, a best practice approach is still lacking while clinical measures of WSV in most GI parameters are unavailable. In this context, we aimed to establish a framework to assess the impact of propagating various sets of physiological WSV, as well as using BSV as a surrogate for WSV, and to develop a pragmatic workflow to estimate the plausible WSV in GI physiology parameters that would describe more realistically the observed variation in the PK parameters. This allowed us to identify the WSV associated with GI parameters that could be ‘excluded’ due to incompatibility with observed WSV of PK markers of BE shown in a replicated study.

## METHODS

**Figure 2:**
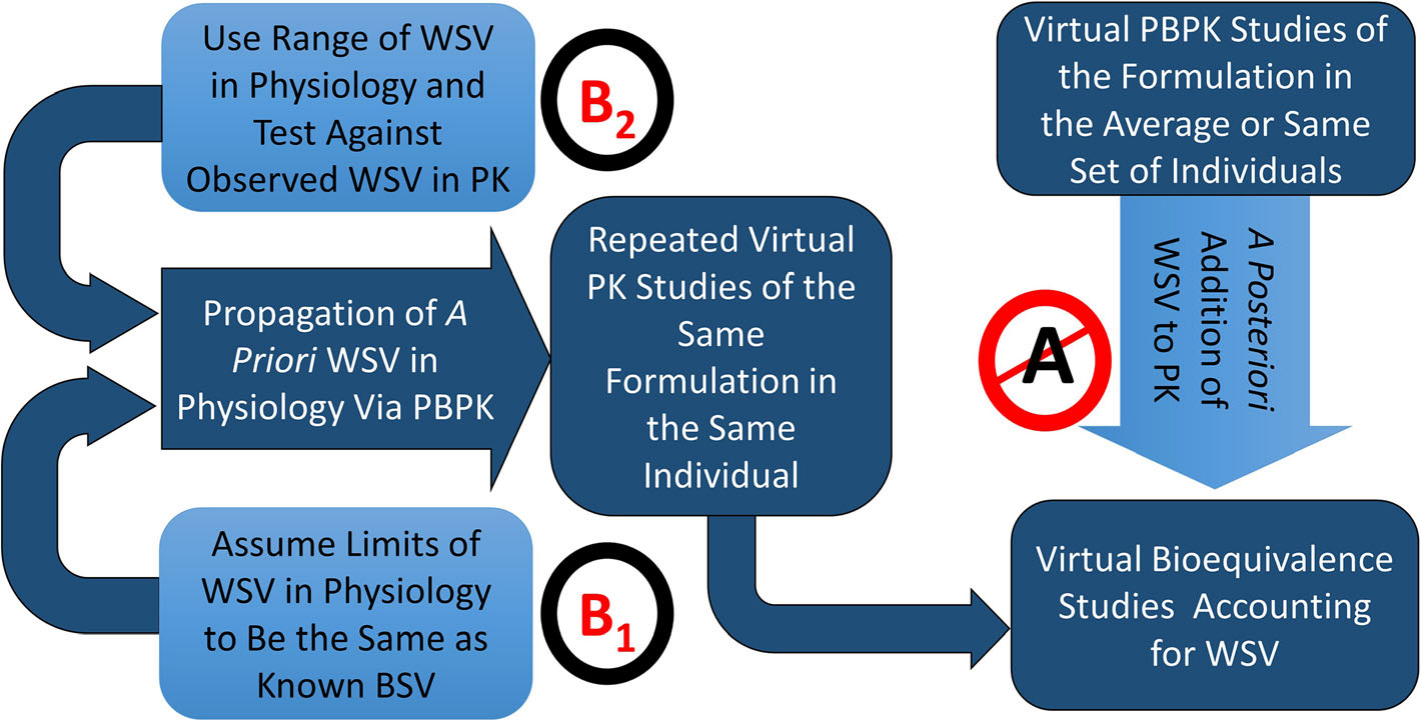
Workflow for the conduct of Virtual Bioequivalence (VBE) studies that accounts for within-subject variability (WSV)

Currently, one of the approaches for VBE is that the WSV in *C*_max_ and *AUC* is incorporated empirically to the PBPK simulations on a *post hoc* way (Path A in Figure 2). This pathway ignores that different formulations may react to WSV of physiology in different ways leading to different WSV for pharmacokinetic metrics. Utility of such approach is limited when we do not know WSV in PK parameters of a given formulation from an earlier study. This path is not ‘ideal’ for predictive work. In this context, the two approaches described herein attempt to establish a framework to simulate WSV in the PK for a given formulation where no prior clinical information on WSV is available. The first modelling strategy assumed the limits of WSV in physiology to be the same as known BSV (Path B1 in Figure 2). Alternatively, we propagated an array of WSV in physiology (Path B2 in Figure 2) and eliminated the sets that are incompatible with observed WSV in PK for a model drug reference formulation from a replicate BE study. The latter involves examining various sets of physiological WSV and exploring the parameter space that is concordant with WSV for PK manifestation of propagating physiological WSV values. This paper applies the proposed strategy on a single model drug/formulation.

### Path B1: Use of Available BSV in Lieu of WSV in Physiological Parameters as a Conservative Measure

WSV in physiological parameters for PBPK model is not yet available for most of the GI tract and other disposition-related parameters. When the purpose of PBPK modelling is to understand the risk of BE failure between the two formulations, a sufficiently verified PBPK model (8) could be used to simulate cross-over VBE trials using hybrid WSV coefficients. Population-based mechanistic PBPK models typically include well-established covariate models based on historical data on human physiology to generate virtual subjects as close as possible to real subjects (25). There are established relationships between physiological parameters (e.g. organ sizes and blood flows) and common demographic parameters such as age, gender, body weight and height of subjects.

One such approach is implemented as ‘Fixed Trial Design’ option of a PBPK platform, Simcyp simulator. Demographics (age, gender, body weight and height) of each of the subjects recruited in a clinical study design could be used to define virtual volunteers for virtual crossover BE via this approach. PK sampling time points matching the clinical trial design can be chosen to output the simulated PK profiles from including definition of limit of quantification (LOQ) to mimic clinical study as close as possible. Two trials of this set of subjects can be simulated for both the formulations. As the age, gender, body weight and height are same in each of the 2 trials for a given subject, the corresponding physiology parameters known to be a function of these covariates such as tissue volumes and blood flows would be same between the two trials. However, parameters such as gastric emptying rate or pH of GI tract segments that are not a direct function of defined covariates will be generated from the mean and %CV defined for the parameter in the PBPK platform. The mean and %CV of parameters defined in the Simcyp Simulator are mainly derived from groups of subjects so they represent BSV rather than pure WSV. In the absence of clinical measures of WSV in GI physiology parameters, BSV available in the Simcyp Simulator can be assumed to generate the hybrid WSV for those parameters.

Since BSV generally exceeds WSV levels, this approach may inflate the sponsor risk/type II error in BE (i.e. false negative BE). Alternatively, simulating virtual twins of individual subjects multiple times may mitigate the inflation of WSV by using BSV as a surrogate. When simulating virtual twins, parameters outside the domain of the built-in correlated Monte Carlo algorithm would likely have more similar values across twins, since they are based on a pseudorandom sequence number. However, besides being time consuming, generating virtual twins may also artificially underestimate the amplitude of WSV in PK metrics and hence, increasing type I error/patient risk (i.e. false positive BE results).

### Path B2: Propagating an Array of WSV in Physiology and Eliminating the Sets Which Are Incompatible with Observed WSV in Pharmacokinetics

Overall, the WSV coefficient derived in 2×2 crossover designs is a hybrid parameter, lumping PK variability related to test and reference formulations. On the other hand, partial or full replicated designs (RTR/RTRT) assess formulation-dependent WSV and thus, modelling such data may offer a unique opportunity to identify WSV descriptors. After developing and validating a PBPK model for a model drug, we carried out local and global sensitivity analyses to identify the GI parameters that are influential for the systemic exposure of the selected model drug. It should be noted that for some other drugs and formulations, these sensitivities would be different, although the underlying physiological variabilities will be largely independent of the drug and formulation, unless the drug itself is acting on the GI tract physiology.

An array of different combinations of WSV in the most influential GI parameters was propagated through the PBPK model of the selected drug. Simulated WSV of drug exposure parameters for the various combinations of GI WSV was compared to the observed WSV obtained from the clinical RTR BE study in order to eliminate the combinations that were incompatible with the observed.

#### Clinically Observed WSV in PK

The following selection criteria were applied to interrogate the BE database from the Croatian Drug Regulatory Agency^1^ in order to identify replicated BE studies for an optimal model drug to illustrate this modelling strategy: active substance of known high WSV, sufficiently large sample size to allow comparison of distribution frequencies, and previously developed oral absorption model for the selected compound. Importantly, the clinically observed WSV in PK will not be used as an input, but to validate this modelling strategy.

The selected study had a partial replicated design (RTR) with a sample size of 66 healthy male subjects (18–45 years, BMI 18.5–30 kg/m^2^), who were randomized for this open-label, two-treatment, three-period, three-sequence, single oral dose cross-over, partial replicate BE investigation under fasting conditions. Sixty subjects had evaluable PK on both occasions. The substance posaconazole was known for demonstrating high WSV, being a poorly soluble weak base and highly permeable molecule (BCS II). The formulations investigated were gastro-resistant tablets of the reference and the test product. Study protocol was approved by the Independent Ethics Committee.

For a subset of individuals (*n*=9), variation between the two occasions for the complete concentration-time profiles was assessed graphically by plotting the variation in concentration at each time point *vs* the average profile to detect any consistent patterns. WSV was estimated as:

1 $$Measure\ of\ WSV=\frac{\left|\Big( PK\ {Parameter}_{Occasion\ 1}- PK\ {Parameter}_{Occasion\ 2}\right)\Big|}{Mean\ \left( PK\ Parameter\right)}$$

Data analysis revealed no formulation, period or sequence effects. Thus, the two occasions for administration of reference product could be treated equally regardless of the sequence and period.

### Simulated WSV in PK

#### Development of a Full PBPK Model

The reference drug product for this modelling exercise is a delayed-release formulation; therefore, the modules for enteric-coated tablets with triggering pH were selected in the simulator (26). Human effective permeability (*P*_eff_) was estimated from available *in vitro* data, using metoprolol as a calibrator (27). Renal elimination was considered negligible and was not included in the model. A simplified elimination parameter in the form of intravenous clearance was used. Table I shows the input parameters used in the PBPK modelling.

**Table I:** Parameter values used for model drug in the Simcyp Simulator

Parameters	Model drug	Reference/comments
Formulation		
Dosage form	100 mg enteric-coated tablets	Gastro-resistant tablets (28)
Triggering pH	6.5	[(28)]; value refined according to the observed data
Phys-chem		
Molecular weight (g/mol)	700.8	[(5, 6, 29)]
LogP	4.6	[(5, 6, 29)]
Compound type	Diprotic base	[(5, 6, 29)]
pKa1	3.6	[(5, 6, 29)]
pKa2	4.6	[(5, 6, 29)]
Absorption	ADAM	
*P*_eff_, man (10^−4^ cm/s)	2.17	Predicted using Caco-2*P*_app_-*P*_eff_ correlation model in ADAM
*P*_app_, Caco-2 (10^−6^ cm/s)	12	[(27)]; reference compound metoprolol
Critical supersaturation ratio	7.3	[(29)]; HPMCAS protects against precipitation, supersaturation sustained
Precipitation rate constant	4	[(29)]
Intrinsic solubility (mg/mL)	0.001	[(5)]
Distribution	Full PBPK	
*V*ss (L/kg)	2.54	[(5)]; Estimated using Rodgers and Rowland method with Kp scalar of 0.06
fu	0.02	[(5, 6, 29)]
*B*/*P* ratio	1.14	[(5, 6, 29)]
Elimination	*In vivo* IV clearance	
CL (IV) (L/h)	4.8	[(30)] reported 6.54 L/h; value refined according to the observed data

**Figure 3:**
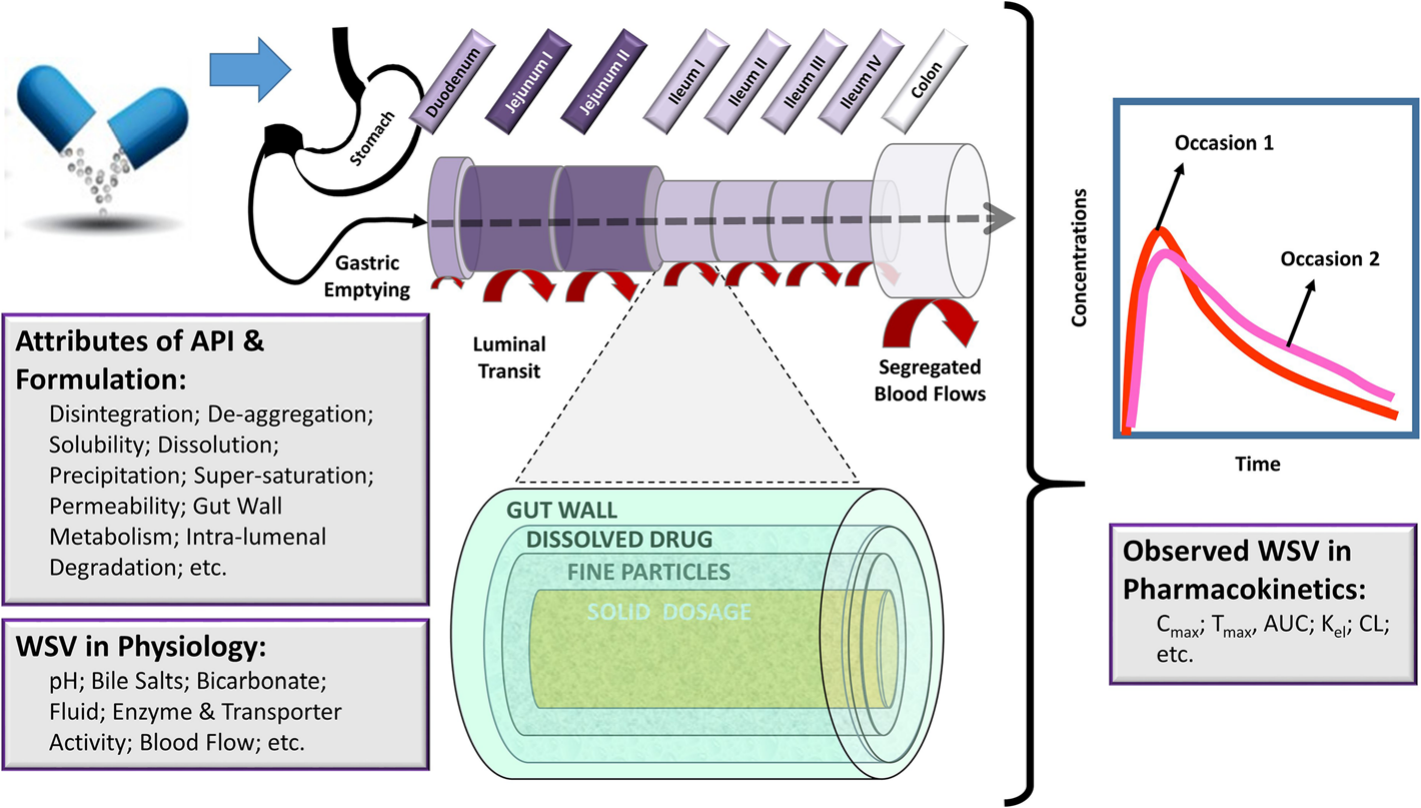
Propagation of WSV in the physiological attributes of the GI tract through the interaction with attributes of the API (Active Pharmaceutical Ingredient) and the formulation within a mechanistic model representation of oral absorption (in our case ADAM Model of Simcyp™ Physiologically-Based Pharmacokinetic Simulator)

The *in vivo* dissolution rate of drug was mechanistically estimated based on a diffusion layer model (DLM) assuming a spherical particle with a non-linear decrease in dissolved drug concentration when moving away from the surface (Wang and Flanagan equation) (31). Monodispersed particle size distribution was used with particle radius of 10 μm and dissolution scalar equal to 1. The DLM allows to account for regional differences in GI tract, BSV as well as WSV in physiology (luminal fluid volumes, pH, bile salt levels, etc.). See Figure 3.

Model performance was assessed comparing simulated and observed PK metrics (*C*_max_, *T*_max_ and *AUC*). Virtual population mimicked the sampled group in the clinical trial, i.e., 66 healthy subjects and age range 18–45 years. Model adequacy was concluded when simulated values were within twofold of their observed counterparts. (Please see Supplementary material.)

#### Selection of GI Physiological Parameters for Propagation of WSV Based on Sensitivity Analysis

Both local and global sensitivity analyses were carried out to identify the GI parameters that are most influential for the developed PBPK model. The outcome metrics for the sensitivity analysis were *C*max, *AUC* and *T*max.

Briefly, we assessed the impact of the following parameters using sensitivity analysis before engaging with propagation of any WSV to PK:

- Initial volume of stomach fluid
- Luminal pH (in stomach, duodenum, jejunum I and jejunum II)
- Luminal mean residence time (in the stomach, small intestine and colon)
- Duration of the IMMC (interdigestive migrating motor complex) cycle
- Bile salt levels (in duodenum, jejunum I and jejunum II)
- Bicarbonate levels (in duodenum, jejunum I and jejunum II)

The values of the selected parameters were varied within their physiologically relevant range. It can be expected that, physiologically, values for many of those parameters will be correlated to some extent, e.g. pH in the stomach will most likely be higher if the initial volume of stomach fluid is larger, or there will likely be some correlation of the pH across the different intestinal compartments. However, these correlations are yet to be quantified. Thus, these could not be implemented in the sensitivity analysis.

Following the initial step of conducting local sensitivity analysis, the global sensitivity analysis using the Morris method was carried out based on influential parameters of the first step (32).

#### SIMULATIONS of WSV in PK and Comparison with the Observed WSV in PK

A set of virtual twins was created in the Simcyp simulator.

Various combinations of the variability assigned to the selected GI parameters were first investigated in an exploratory phase. This analysis showed that the model output was sensitive to the changes in CV% of the GI parameters and that method could be used for testing different permutations of the GI parameter variability in the full set of virtual individuals. Moreover, since clearance of this model drug is not highly variable within the same individual, based on the observed data, its CV% was approximated to 5%. Within the same individual, variation in volume of distribution is known to be less than variation in clearance (33); therefore, the observed inter-occasion changes in the elimination rate constant were attributed to CL variability. Even though for some subjects there was zero change in the *k*_el_ value, for others the change was notable. Therefore, WSV for CL was also propagated through the simulations. CVs% for liver volume and brain volume were set to zero to simulate the same liver and brain weight on multiple occasions in the same individual. Kidney weight was already set to simulate the same values. PK sampling time points were mimicking the time points of the actual BE study up to 120 h.

Following the exploratory phase of investigating the effects of changing variability of the GI parameters on the PK profiles, a full set of virtual subjects was produced mimicking the 60 subjects enrolled in the clinical trial, who had evaluable PK on both occasions. Every subject was simulated at two occasions only, and a random seed was selected.

Screening of the parameter variability space was performed on the full set of virtual twins (*n*=60) by assessing the different combinations of CV% for the selected GI parameters (Table II). Simulations started by using the default simulator CV% values, presenting the likely BSV values for various GI parameters. It is expected that WSV should be lower than BSV values of the simulator. Therefore, all other combinations tested only lower CV% values than default. Since simulations of each virtual subject for each scenario were processed manually, only a limited number of scenarios was investigated and the aim was to detect the most sensitive ones. Therefore, CV% were first reduced by half. Reduced levels of variability were implemented on several grouped parameters (e.g. intestinal pH and bile salt levels) in different combinations, on all tested parameters or on a single parameter, as shown in Table II. The intent was not trying to identify the most plausible WSV scenario, but rather to eliminate the most improbable ones.

**Table II:**
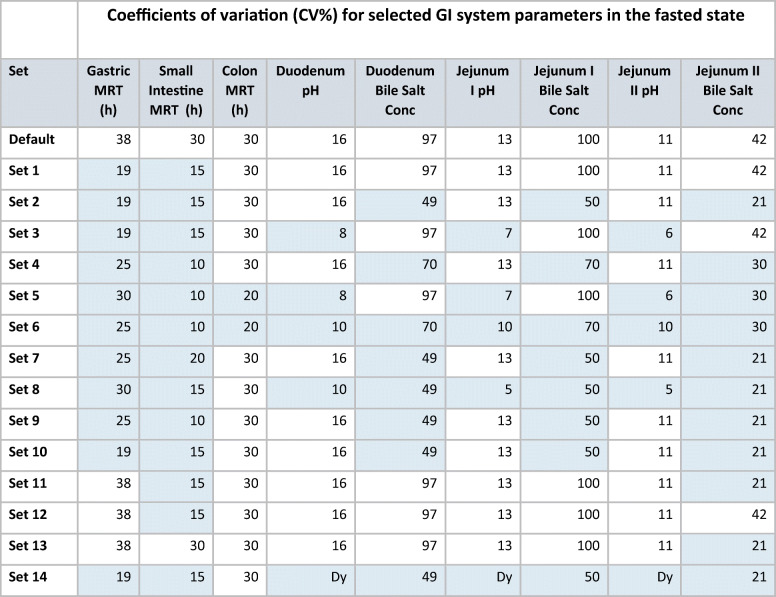
Different sets of WSV in GI physiology investigated

CV% differing from the default (BSV) value are highlighted in blue. CV% for volume of water administered was set to 1%, initial volume of stomach fluid at 30%, stomach pH at 38% and drug clearance to 5% in all sets except Set 10 (where CV% in CL was set to 0%). CVs for liver and brain volume and kidney weight were set to zero. Dy, dynamic (option in the Simcyp simulator)

A separate workspace was produced for each individual and each combination investigated. All workspaces for one combination were run at once using the batch processor. Variability in the simulated *C*_max_, *T*_max_ and *AUC*_0-*t*_ for each individual was calculated the same way as for the observed data (Equation 1). Cumulative frequency distribution of the simulated variability in PK parameters was made to allow its comparison to the observed distribution of the variability.

A two-sample Kolmogorov-Smirnov test was used to examine the similarity between two distributions (*p*-value < 0.05). Goodness-of-fit of the simulated variability distribution was evaluated by ranking of the calculated D values and by visual inspection of the graphical outputs of the cumulative frequency distribution curves.

#### Virtual Bioequivalence

The demographics of 66 subjects who participated in the clinical study (age, gender body weight and height) were used to define virtual subjects via ‘fixed trial design’ of Simcyp simulator and 10 virtual trials were simulated for reference product using default (Path B1, Figure 2) and SET2 (Path B2, Figure 2) physiology (Table 2) options. WSV was mechanistically propagated in the simulations to predict variability in the PK exposure measures (VBE of R *vs* R). Average bioequivalence was evaluated in Phoenix WinNonlin (v8.3; Certara; Princeton, NJ, USA) for parameters *AUC* and *C*_max_. BE was calculated using each of the 10 trials as a reference while treating remaining 9 trials as test. From the clinical PK profiles of reference product across the two repeat periods, BE was calculated between period 1 and period 2; each of the 2 periods was used as a reference while treating the other period as test. Then the default (Path B1, Figure 2) and SET2 variability (Path B2, Figure 2) based simulations were compared to clinical PK data.

Additionally, simulations were performed for SET2 physiology using smaller sample sizes (*n*=12, 24 and 48). Clinical data was also reduced in sample size by ten random sampling of 12, 24 or 48 subjects from the initial sample of 66 subjects. Ten virtual trials were made for each sample size and VBE was calculated between period 1 and period 2 for each of the ten trials; each of the two periods was used as a reference while treating the other period as test. Then the simulated data for each sample size were compared to clinical PK data.

## RESULTS

### Propagating an Array of WSV in Physiology and Eliminating the Sets Which Are Incompatible with Observed WSV in Pharmacokinetics

#### Observed WSV in Pharmacokinetics

Judged by the inter-crossing of the lines for the absolute difference concentration from the average of two occasions (see ‘Methods’), the biggest variation of PK profiles was observed within the first 5 to 6 h, which approximates to the respective *T*_max_ values. Figure S2 (Supplementary material) shows a typical example from the subjects examined.

**Figure 4:**
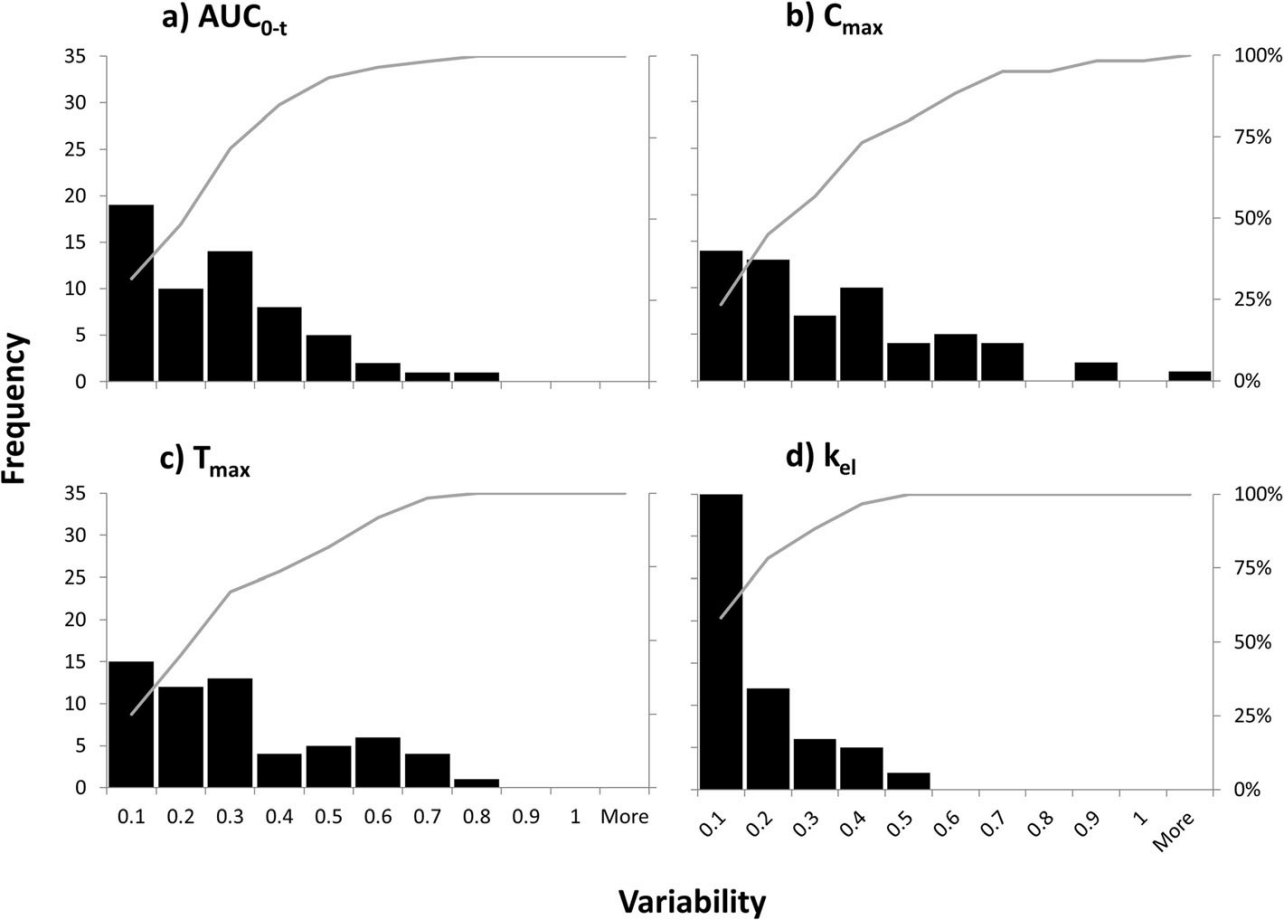
Cumulative frequency distribution of the observed variability in *AUC*_0-*t*_, *C*_max_, *T*_max_ and *k*_el_ (60 subjects with evaluable PK from RTR BE study)

Cumulative frequency distributions of the observed variability in some PK parameters (*AUC*, *C*_max_, *T*_max_ and *K*_el_) are shown in Figure 4 and demonstrate the highest WSV being associated with *C*_max_, while the lowest WSV was related to first-order elimination rate constant.

#### Simulated WSV in Pharmacokinetics

The developed full PBPK model showed an adequate fit to the observed clinical data (see Figure S1 in the Supplementary material).

PK parameters that define the rate and extent of exposure showed sensitivity to the initial volume of fluid in the stomach, pH in the intestines (but not in the stomach), mean residence time in the GI tract and jejunal bile salts levels (all expected considering the nature of the formulation). GSA allowed ranking of the input parameters with respect to the magnitude of their impact on the model output. The highest impact on *AUC* was observed from intestinal pH (duodenum>jejunum 1>jejunum 2), colon and small intestinal transit time. Intestinal pH (duodenum>jejunum 1>jejunum 2) also had a highest effect on *C*_max_, followed by bile salt concentrations in jejunum 2 and residence time in the small intestine. For *T*_max_, the most influential variables were mean gastric and mean small intestinal residence time, followed by duodenal pH (see Figure S3 in the Supplementary material).

**Figure 5:**
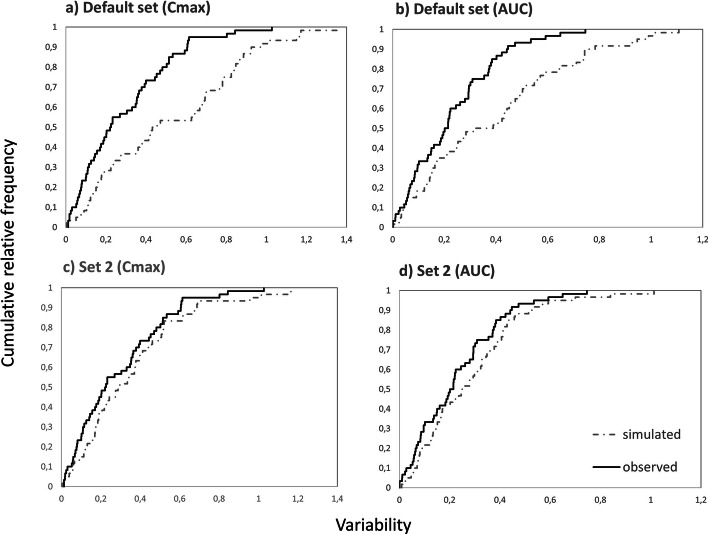
Cumulative frequency distributions of simulated within-subject*C*_max_ and *AUC* variability (using 60 virtual twin subjects simulated for two occasions and mimicking conditions of BE study) for different sets of WSV in GI physiology that showed better (Set 2) or worse fit (Default set) to the observed distributions (see also D statistic in Table III)

Table III shows the level of (in-)consistencies between the distribution frequency of simulated and observed WSV for various PK parameters. According to the Kolmogorov-Smirnov test, assumptions associated with WSV in the default set (i.e. using default BSV in lieu of WSV of GI physiological parameters and low WSV of CL with CV = 5%) produced the most dissimilar frequency distribution of the WSV of the selected PK parameters when comparing the simulated and observed data (*C*_max_: *D*=0.350, *p*=0.001; *AUC: D*=0.417, *p*<0.0001). All other combinations with assumed lower WSV of physiological parameters had better outcome though in many cases, they were still dissimilar to the observed frequency distributions of the observed variability in some PK parameters (e.g. *AUC*: default, sets 3, 5, 12 and 13; *C*_max_: additionally sets 6 and 9). In general, this combinatorial approach allowed us to identify series of settings where the simulated WSV of the physiological parameters were inconsistent with observed WSV of *C*_max_ and *AUC*. Interestingly, no inconsistency between observed WSV in *T*_max_ and simulated values could be discerned. Figure 5 shows an example of best and worst consistencies between the distribution frequency of simulated and observed WSV for parameters *C*_max_ and *AUC*.

**Table III:**
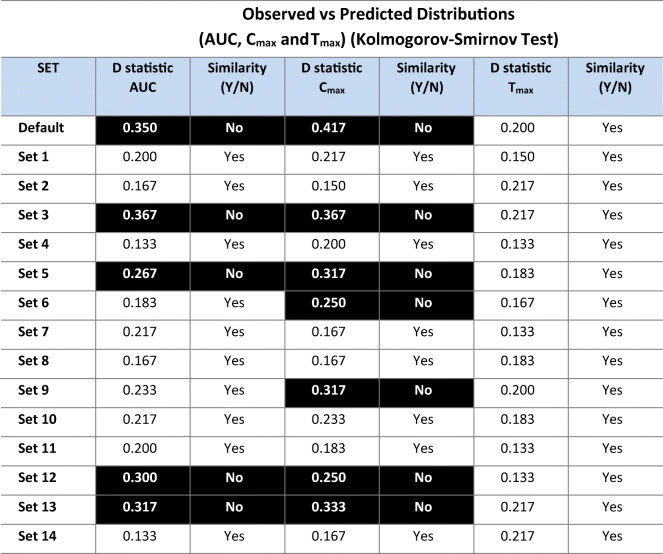
Results for the comparison of simulated *vs* observed within-subject variability (set of 60 individuals, Kolmogorov-Smirnov test)

Two-sample KS:

*p*-value > 0.05 = null hypothesis cannot be rejected (two samples follow the same distribution)

*p*-value < 0.05 = null hypothesis should be rejected (the distributions of two samples are different)

Hence, highlighted cells, WSV Model Not Supported

#### Virtual Bioequivalence Studies of Reference *vs* Reference Product

**Figure 6:**
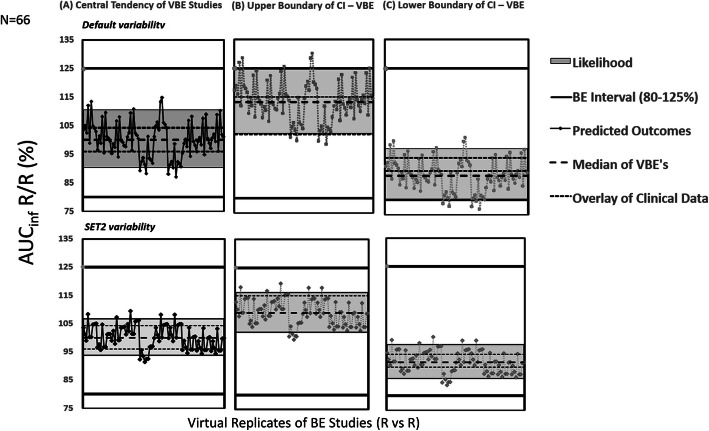
Virtual replicates of bioequivalence studies (R *vs* R) for *AUC*_inf_: upper row shows results for propagation of BSV as WSV (Method B1), lower row shows results for propagation of SET2 variability as WSV (Method B2); overlaid with observed clinical BE results

Results showed that the SET2 physiology (Path B2) is capturing the clinical PK data of reference product closely while default BSV assumed as WSV (Path B1) produced inflated 90% confidence intervals (Figure 6).

It is important to note the median R/R ratios of default, SET2 and clinical PK are around 100% thus showing the central tendency of reference product being detected as reference is achieved in all cases. However, the 90% CI which represents the impact of WSV on the BE outcome is strongly affected by what WSV was considered during simulations. Similar, however, not so obvious trend was observed for *C*_max_ parameter (see Figure S4 in the Supplementary material).

**Figure 7:**
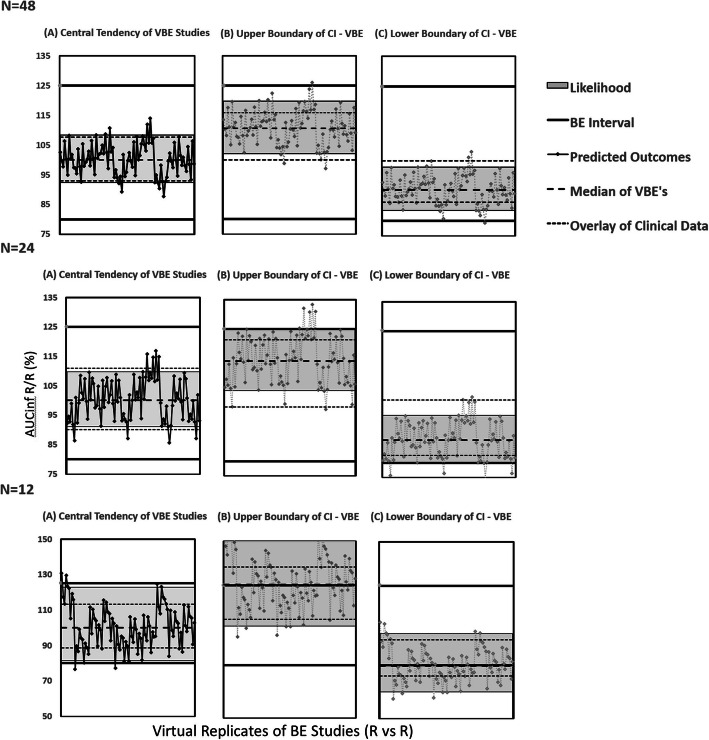
Virtual replicates of bioequivalence studies (R vs R) for *AUC*_inf_ using SET2 variability as WSV, with different sample sizes (*n*=12, 24 or 48); overlaid with clinical BE results

To further test the ability of SET2 physiology to mimic clinical data, additional simulations with reduced sample size (*n*=12, 24 and 48) were performed. The simulation with smaller number of subjects also showed a reasonable alignment of simulated and clinical data (see Figure 7 and Figure S5 in the Supplementary material).

## DISCUSSION

### WSV of PK Parameters: a Dependent Variable

Throughout this report, we have tried to make a distinction between the WSV of PK parameters and WSV of physiological and biological attributes. It is important to realize that physiological WSV determines the observed WSV in PK. However, the notion of ‘highly variable drugs’ (/formulations) might have distorted the distinction between these two elements by giving the false impression that the high variability is intrinsic to the drug (/formulation) rather than explaining that some physiological parameters are highly variable within the same subject but these may affect some drugs (formulations) more than the others.

Separation of WSV and BSV is possible by applying mixed-effect models and through replicate studies (34). However, replicate studies designed to measure the WSV in physiological parameters and application of mixed effect modelling to analyse data outside the PK and PD domains are rather rare. Considering the numerous physiological factors of GI tract affecting oral drug absorption, direct assessment of all of them experimentally seems very unlikely in near future. Indeed, even the population studies designed to assess hybrid metrics of WSV and BSV for GI parameters are also lacking in many cases.

In the simulator, many of the GI physiological parameters that are not known to co-vary with demographic parameters are described by their mean value and distribution (CV%) known from multiple clinical studies measuring that physiological parameter, e.g. gastric emptying rate. Thus, the measure of variability (CV%) is a mixture of WSV, BSV and residual variability. In this work, we were aiming to find plausible combinations of WSV of sensitive physiological parameters. Further separation of random residual variability from WSV would be even more challenging. Left-overrandom variability is considered to be spread across BSV and WSV space. Once we repeat this exercise on a wider set of drugs and formulations in the future and arrive at a common set of WSV parameters that explains wider range of PK datasets, pharmacometrics approaches such as non-linear mixed effect modelling can be employed further to fine-tune the sources of variation, including residual variability.

In the absence of WSV of the physiological parameters, one approach (Path A) in the field of VBE has been arbitrarily assigning WSV to simulated PK parameters based on previously observed values in clinical studies. A major limitation of such approach is that it ignores the fact that WSV in PK is a dependent parameter defined by the interaction of the formulation and the drug with WSV of the physiology (see Path A in Figure 2).

### WSV of Physiological Parameters: Mostly Unknown but Possible to Estimate

Despite the fact that majority of WSV values for physiological parameters are not known, it is possible to discern the most likely boundaries for each via independent studies where the outcome measure is sensitive to certain WSV in physiological parameter (see Figure 3). Assuming the PK outcome is sensitive to a given physiological attribute, overestimation of WSV in the physiological parameter will lead to overestimation of the WSV in observed PK outcome whereas underestimation of WSV in physiology would be associated with underestimation of the WSV in observed PK parameters.

Different drugs and formulations will have different sensitivities to a range of physiological parameters. Hence, the results shown for the sensitivity analysis in this study are unique to the drug and specified formulation. Therefore, delineating the WSV of physiology based on WSV of PK outcome measures involves solving several equations (several drugs/formulations) with several unknown parameters (WSV of physiology).

Replicate BE studies provide a golden opportunity to obtain and use the WSV of PK measures for estimating the intrinsic WSV of physiology that are common to well-controlled BE studies.

In this proof-of-concept study, we simulated VBE trials for 12, 24 or 48 virtual subjects and compared the amplitude of the simulated 90% CIs for *C*_max_ and *AUC*_inf_ to the respective observed counterparts calculated using the same truncated sample size and randomly selecting individuals from the pool of subjects enrolled in the clinical BE study. Conceptually, we have emulated the subjects sampling process and recreated multiple plausible *in silico* and *in vivo* BE studies for the model drug. The goal was to verify the WSV model rather than focusing on statistical power of the VBE study.

### Stepwise Exclusion of Improbable WSV of Physiological Parameters

**Figure 8:**
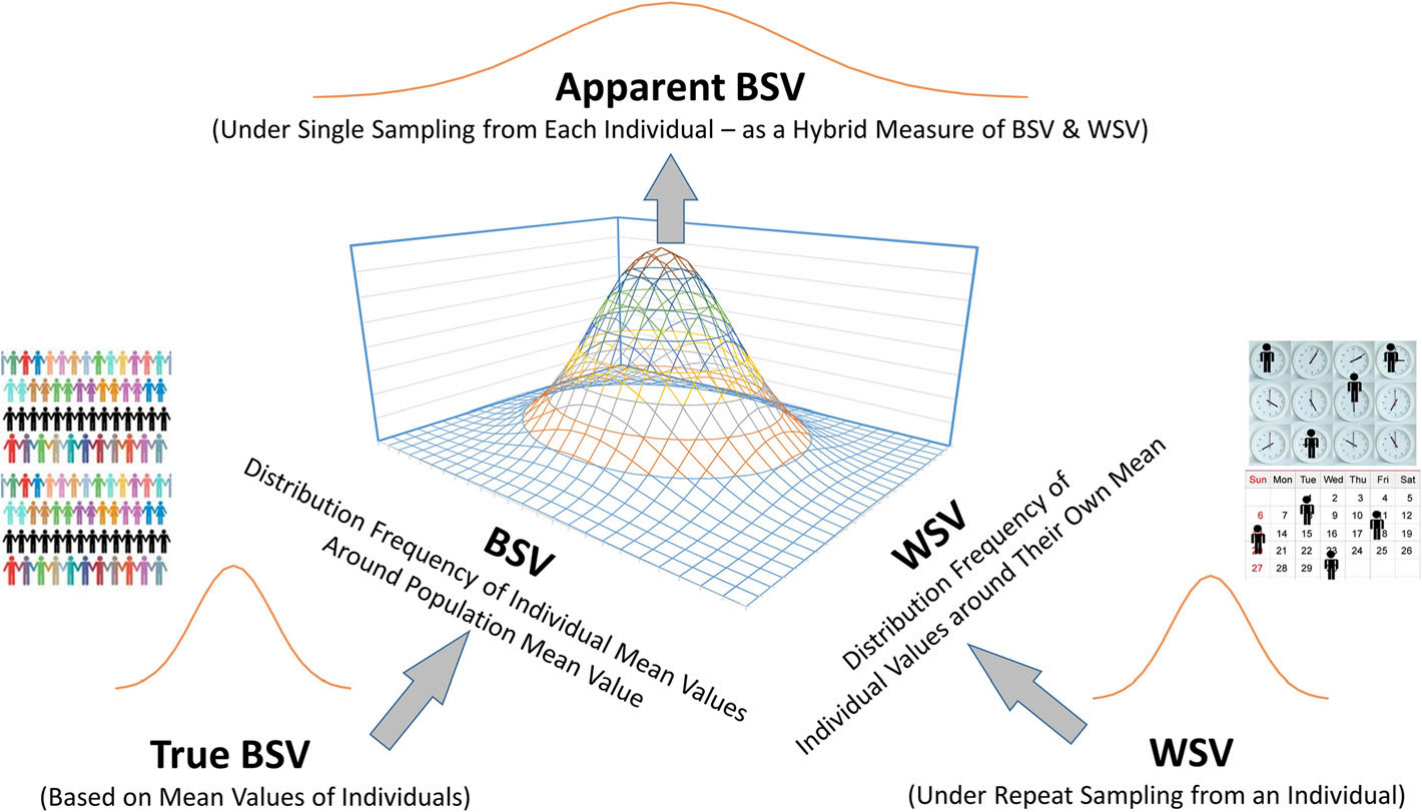
Visualization of the relationship between the true BSV and WSV and the manifestation of apparent BSV when only a single measurement is obtained from each individual in the population that does not capture the individual mean. Apparent BSV in these cases would seem wider than actual BSV and the degree of inflation will depend on the extent of WSV of the parameter. No measurement error (residual variability) is assumed, otherwise that is also added to all distributions.

Once a suitable drug (/formulation) with sensitivity to certain physiological parameter(s) is identified, the most logical first step in assigning WSV to physiology for propagation to PK through PBPK modelling would involve using the hybrid value of WSV and BSV. Such parameter values are known in many cases albeit with the misnomer of ‘BSV’ applied to them instead of indicating the hybrid nature due to lack of mixed effect modelling. The concept is shown visually in Figure 8.

Since such hybrid measures of BSV/WSV are generally greater than WSV, this approach may inflate the amplitude of the estimated 90% CI’s for exposure metrics. The simulated VBE outcomes shown in Figure 6 demonstrate this fact. However, from the risk assessment perspective, these will likely provide worst-case scenario for WSV impact on BE outcome of the two formulations.

The alternative would be to assign lower WSV than known values for hybrid WSV/BSV and examine their performance against observed WSV of PK parameters. Importantly, this approach is not intended to reduce the variability of simulated PK metrics, but to create compatible variations in the simulated scenario that could likely explain WSV of observed PK metrics.

We followed this approach but as it was expected while several sets of WSV for physiological parameters could be dismissed (Table 2), there were several sets that could provide reasonable consistency regarding the WSV of outcome measures (PK parameters). Therefore, we could exclude certain values for WSV of physiological parameters under the typical BE study design (and not specific to this drug or formulation) but narrowing down the remaining sets will require similar exercise using other drugs and formulations which demonstrate different pattern of sensitivity in the PK outcome in relation to the physiological parameters of GI tract.

In this study, the array of WSV values intended for exploration was chosen empirically due to mostly manual processing and rather time-consuming simulation for each combination/individual. Any future studies could benefit of systematic and automated assessment of the complete WSV parameter space. PBPK platforms are also evolving to support such analysis with further automations.

## CONCLUSION

The current report, for the first time, provides a modelling approach that enables estimation of the WSV of physiological parameters of the GI tract, which are essential for conducting robust crossover VBE studies.

Since we carried out the study only for one drug and a single formulation, we were able to eliminate only certain sets of WSV of physiological parameters that this particular drug and formulation were sensitive to.

Nonetheless, the feasibility of the strategy is demonstrated. Extending this work to other drugs and formulations would enable in narrowing the parameter space related to WSV values for sets of physiological parameters, which can be used ‘prospectively’ for predicting the VBE with realistic estimates of CI around any given formulation. With increased confidence in established WSV, verified on larger PK data sets, the PBPK model can be used to estimate power of BE studies, estimate optimal sample size and inform study designs.

## Supplementary Information

ESM 1 (DOCX 1067 kb)

## Footnotes

1 Agency for Medicinal Products and Medical Devices (HALMED)

## Declarations

### Disclaimer

Views expressed in this article are personal opinion of the authors themselves, formed by their scientific judgement, and do not reflect policies, view or required practices of affiliated organizations.
