# Supplementary material for: Proof of Concept in Assignment of Within-Subject Variability During Virtual Bioequivalence Studies: Propagation of Intra-Subject Variation in Gastrointestinal Physiology Using Physiologically Based Pharmacokinetic Modeling
Source: AAPS J. 2022 Jan 5;24(1):21. doi: 10.1208/s12248-021-00672-z (PMC8817238; doi:10.1208/s12248-021-00672-z)
Supplement: Supplementary file 1 — (DOCX 1067 kb) [file 12248_2021_672_MOESM1_ESM.docx]

**Figure S1** Graphical evaluation of model fit (predicted vs observed, semilog plot). Lines represent median, 5th and 95th percentiles. Observed PK profiles are following the central tendency and are captured well within 5th and 95th percentile of the simulated profiles.

**Figure S2** Single occasion relative to the mean of the two occasions for observed full PK profiles for a subset of selected subjects (n=9). The biggest variation between the two occasions is observed within first 5 to 6 hours (black diamond); for the rest of the profile, the variation was generally lower than 0.2-fold from the mean. Most inspected profiles were similar to example A, with one occasion clearly above the average and the other below the average value. Example B shows a subject with multiple crossing of the two occasions with their average.


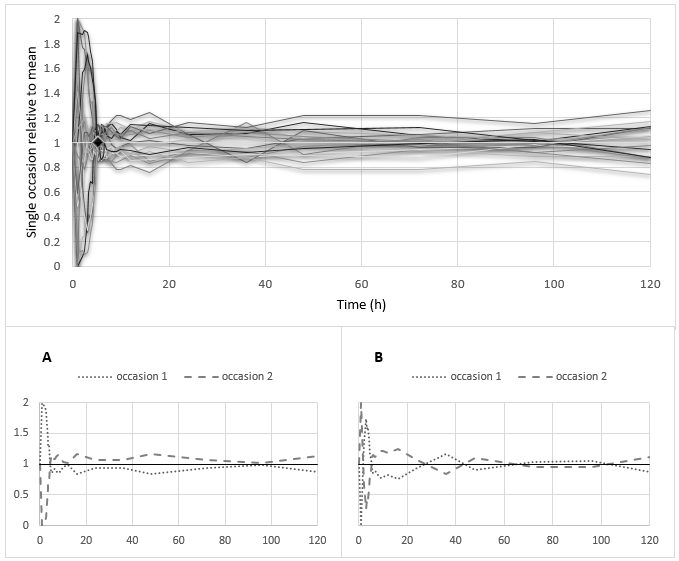


**Figure S3** Results of global sensitivity analysis (Morris method) ranked per the absolute mean (μ*) values. High μ* indicates a parameter with an important influence on the model output (AUC, C_max_ or T_max_).

**Figure S4** Virtual replicates of bioequivalence studies (R vs R) for C_max_: upper row shows results for propagation of BSV as WSV (Method B1), lower row shows results for propagation of SET2 variability as WSV (Method B2); overlaid with observed clinical BE results


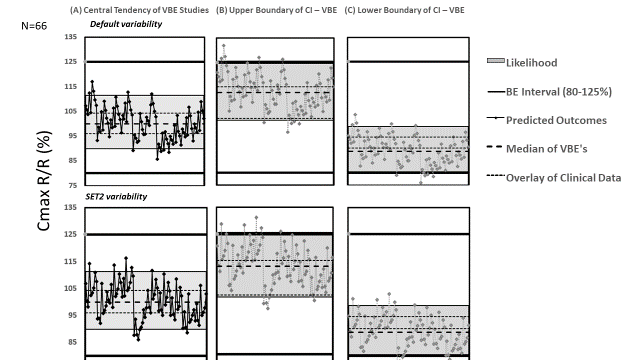


Virtual Replicates of BE Studies (R v R)

**Figure S5** Virtual replicates of bioequivalence studies (R vs R) for C_max_ using SET2 variability as WSV, with different sample sizes (n=12, 24 or 48); overlaid with clinical BE results


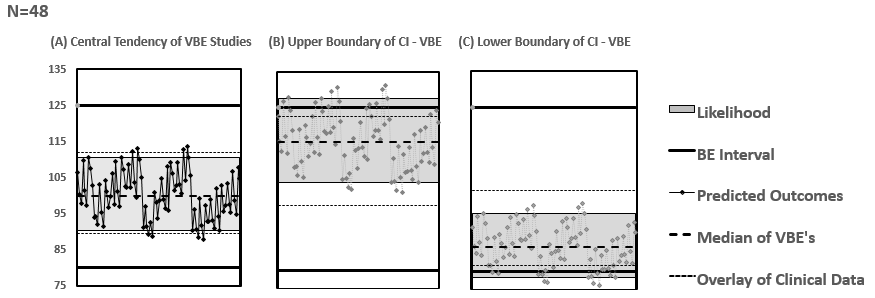


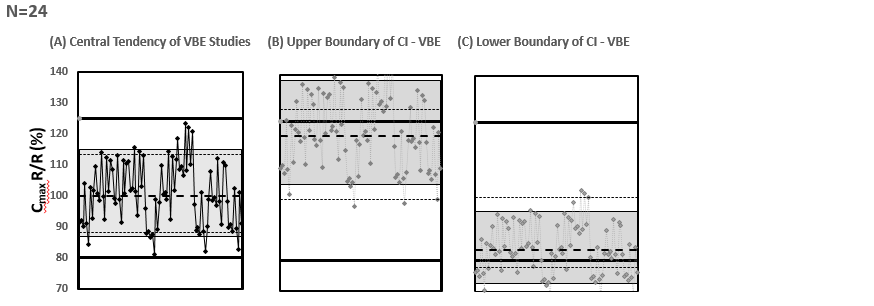


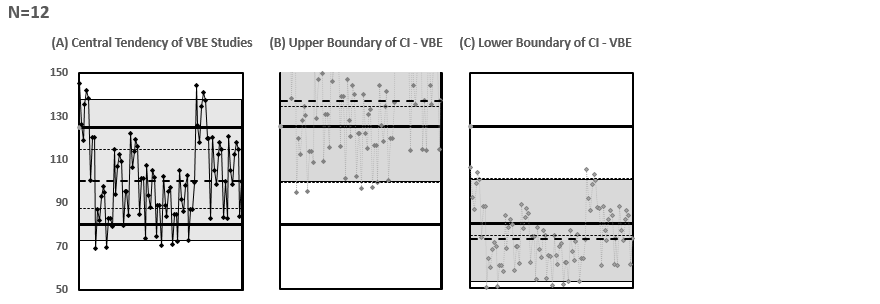


Virtual Replicates of BE Studies (R vs R)
